# Ergonomic workplace analysis (EWA) as a model for creating an instrument to assess rest locations for truck drivers

**DOI:** 10.47626/1679-4435-2023-817

**Published:** 2023-04-18

**Authors:** Felipe Pereira Rocha, Talita Silveira Campos Teixeira, Claudia Roberta de Castro Moreno

**Affiliations:** 1 Instituto de Ciências Puras e Aplicadas, Universidade Federal de Itajubá, Itabira, MG, Brazil; 2 Departamento de Saúde, Ciclos de Vida e Sociedade, Faculdade de Saúde Pública, Universidade de São Paulo, São Paulo, SP, Brazil; 3 Psychology Department, Stress Research Institute, Stockholm University, Stockholm, Sweden

**Keywords:** ergonomics, sleep, truck drivers, ergonomic analysis, ergonomia, sono, motoristas de caminhão, avaliação ergonômica

## Abstract

**Introduction:**

The relationship between sleep quality and rest location is rarely explored in the literature. In this context, ergonomic analysis instruments can contribute with information for a satisfactory rest environment throughout the work schedule.

**Objectives:**

To analyze the performance of an instrument based on Ergonomic Workplace Analysis for assessing rest locations.

**Methods:**

This study adapted an ergonomic instrument to a new purpose. To analyze its performance, we assessed the rest locations of truck drivers working for a large transportation company located in the state of São Paulo.

**Results:**

The variables adapted from the original Ergonomic Workplace Analysis were rest location, sequence of tasks, lighting, noise, indoor comfort, and thermal comfort. Photos and flowcharts were used to better describe the data.

**Conclusion:**

The new instrument was shown to be adequate for assessing rest locations. The drivers evaluated the accommodations more positively than the analyst, and truck sleepers and company accommodations were considered different both by the drivers and the analyst.

## Abstract (pt)

**Introdução:**

A relação entre a qualidade do sono e o local de descanso é pouco explorada pela literatura. Nesse contexto, os instrumentos de análise ergonômica podem contribuir com informações para um ambiente de descanso satisfatório ao longo das jornadas de trabalho.

**Objetivos:**

Analisar o desempenho de um instrumento de avaliação de locais de descanso, desenvolvido a partir da ferramenta *ergonomic workplace analysis* (análise ergonômica do posto de trabalho).

**Métodos:**

O presente estudo se caracteriza por adaptar um instrumento ergonômico para uma nova finalidade. Realizou-se avaliação dos locais de descanso de motoristas de caminhão de uma empresa transportadora de grande porte, localizada no estado de São Paulo.

**Resultados:**

As variáveis adaptadas da versão original do *ergonomic workplace analysis* foram o local de descanso, a sequência de tarefas, a iluminação, o ruído, o conforto interno e o conforto térmico. As fotos e os fluxogramas foram utilizados para melhor descrever os dados.

**Conclusões:**

O novo instrumento se mostrou adequado para avaliar locais de descanso. Os motoristas avaliaram mais positivamente os dormitórios do que o analista, sendo que o local de descanso do caminhão e os alojamentos foram considerados distintos tanto para os motoristas quanto para o analista.

## INTRODUCTION

In various work situations, it is impossible to sleep or rest at home between workdays. In this context, we identified the need for an instrument that allowed a systematic evaluation of rest locations, consisting of an important tool for understanding the reality of a work activity that does not allow workers to rest at home. Among the numerous approaches and instruments that could be used for the analysis of rest locations, we chose the Ergonomic Workplace Analysis (EWA). EWA is an instrument that provides information on the workplace. It is used for evaluating workplaces and allows the comparison of different workplaces where the same activity is performed. Its theoretical framework resides in disciplines related to work ergonomics, with the participation of workers from their perception on the aspects that rule work.^1^ When it comes to the evaluation of rest locations, the current literature presents little research on this subject. However, the quality of rest locations has been discussed, for example, in studies in the areas of aviation and transportation.^2-4^

In transportation, the study by Lamond et al.,^2^ performed with train operators, observed that the mean amount of sleep per night (measured by actimetry) was higher when workers got to rest at home (7.8 h) than when they took turns sleeping on the train (4 h), suggesting greater sleep quality at home. Actimetry is a widely used method for non-invasive assessment of human activity-rest cycles by using an accelerometer, which estimates sleep and wake times via an algorithm. The equipment is worn on the non-dominant wrist and records arm movement, from which it extracts the user’s activity and rest. In aviation, sleep diaries were adopted along with actimetry by Roach et al.^3^ in a study with 301 airplane pilots. The results indicated a lower mean sleep recovery during flights when compared to sleep at home. The study by Roach et al.,^4^ on the other hand, evaluated sleep quality and quantity according to the seat position: upright (20°), reclined (40°), or flat (90°). The best sleep parameters were observed with the 90° position, that is, when the seat resembled a bed the most.

When considering truck drivers, the relationship between sleep quality and their respective rest locations was little studied. There are, however, studies by an Australian group, such as those by Darwent et al.^5^ and Baulk & Fletcher,^6^ which sought to contribute to this investigation. In the study by Darwent et al.,^5^ performed with a sample of 32 drivers, three rest locations were analyzed: their homes, truck depots, and the truck sleeper berth. When comparing periods of sleep according to rest locations, those who rested at home fell asleep on average 46 minutes earlier when compared to those who slept in truck sleeper berths and on average 32 minutes earlier when compared to those who slept at the truck depot.

These results are similar to those found by Baulk & Fletcher,^6^ who analyzed a sample of 37 truck drivers. The option of sleeping at home resulted, on average, in longer periods of sleep, better sleep quality, and lower levels of tiredness in comparison with the truck sleeper. However, obstacles that hindered a better sleep quality at home included noise and family problems. The problems reported at rest locations away from home were temperature, noise, and lack of adequate truck stops. The results by Kecklund & Akerstedt^7^ suggest that sleeping in the truck sleeper does not negatively affect sleep quality or has very little effect to the sleep-wake cycle, even in environments with louder noise.

Although the law regulates rest periods, it does not specify adequate conditions for these locations.^8^ Nevertheless, it is known that a rest location providing minimal comfort conditions contributes directly to sleep efficiency. However, few studies are dedicated to analyze the rest locations of truck drivers. In this context, truck drivers were the professional category whose rest locations were evaluated in this study and will be presented as an example of the application of an instrument based on EWA.

The aim of this study was thus to evaluate the performance of an instrument that analyzed the rest locations of truck drivers and was developed based on EWA. With the obtained results, we intend to characterize the variables that, according to the instrument, lead to a poor evaluation of the rest location, as well as to identify the instrument’s flaws.

## METHODS

This is a methodological study.^9^ In order to analyze the instrument, we assessed the rest locations of drivers working for a heavy truck transportation company located in the state of São Paulo from January to April 2018. The company works with dry shipping and has branch offices in various states of the South and Southeast regions, with a fleet of more than 1,250 light-, medium-, and heavy-duty vehicles. The participants were truck drivers responsible for transporting goods between the branch offices of Campinas and São Paulo (state capital). Research authorization was obtained through contact between the investigator and the company board.

This research proposal was submitted to and approved by the Research Ethics Committee of the Public Health School of Universidade de São Paulo (No. 2,995,488). The participants were informed on the study and on the need to fill a free and informed consent form for participating, being ensured of the confidentiality and voluntary nature of the research.

### EWA

EWA was created by the Franco-Belgian school of ergonomics and stands out for its analysis of three realities: working conditions, the outcome of the work, and the work task as a unit of work activity. According to Guérin et al.,^10^ the task is defined as the results and previously established conditions for the employee’s work organization, that is, it is mandatory, detached from reality, and planned without considering the real variabilities and constraints of the real activity. The work activity concerns the operational strategies elaborated by employees to adapt to the gap between the prescribed work and the inherent constraints of the real work. The outcome of the work as a unit of work activity refers to the results and real conditions that are effectively put into practice.

For Daniellou,^11^ the “dynamics of transformation of constraints” is one of the domains of ergonomics. By considering the prescribed work as actions previously set to workers through rules and obligations, it shows constraints as a result of a context between the collective action developed by workers in the attempt to achieve the results expected by the organization and the rigid conditions structured by the organization.

The construction of a process of ergonomic analysis is characterized by being dynamic, that is, carried out throughout the action. However, a set of phases aims to put an order to the ergonomic action, where the demand is usually the starting point. This way, ergonomic action, according to Guérin et al.,^10^ is built from demands originating from company boards, workers, and their union representatives, professional institutions, or public offices. In the following paragraphs, we present observations that were used for collecting potentially important information for strengthening the workplace analysis, considering the study objective.

After the observations, we had to systematize the obtained information and initiate systematic observation, which comprised defined semistructured interviews, according to Gray.^9^

Semistructured interviews are not standardized and are many times used in qualitative analysis. The interviewer has a list of items and questions to go through, but is not required to use all of them in each interview. The order of the questions can also change depending on the direction the interview takes. Moreover, additional questions can be made, including some that may not have been anticipated at the beginning of the interview.^9^

During systematic observation, semistructured interviews are performed for identifying and understanding, under the worker’s viewpoint, the management instruments adopted by the work organization, the constraints and clarifications of operational strategies elaborated for adapting the prescribed measures to reality when considering the quality of rest.

EWA, as an open model, could analyze other variables that influenced sleep quality. Some of these variables are contemplated at EWA during interviews with workers. Thus, for evaluating the rest locations of truck drivers, we elaborated an analysis instrument based on EWA. EWA is originally from Finland, and its translation was performed by a group that specializes in ergonomics at Universidade Federal de São Carlos, named Ergo & Ação^1^. Since then, studies using EWA have been performed in various workplaces and populations, such as dentists, sugar cane workers, workers who interact with screens, and pharmaceutical industry workers.^12-14^

The variables selected for analysis were classified according to a scale ranging from 1 to 5. A score of 1 was given in situations with no relevant disturbances to rest. Scores of 4 and 5 indicated that the variables may occasionally disturb the workers’ sleep.

## RESULTS

Information referring to the sociodemographic characteristics of truck drivers is presented in Table 1. The prevalence of poor sleep quality reached 71.6% of all assessments. In addition, considering the variable “years as a driver,” there is a trend indicating a worse sleep quality for less experienced drivers.

**Table 1:** Sociodemographic information on truck drivers according to sleep quality

Variables	Sleep quality				X^2^ p-value
	Good		Poor		
	n	%	n	%	
Branch office					
São Paulo	13	56.5	31	53.4	0.80
Campinas	10	43.5	27	46.6	
Employment status					
Outsourced employee	17	73.9	44	75.9	0.85
Employee	6	26.1	14	24.1	
Shift					
Daytime	0	0.0	3	5.2	0.26
Nighttime	23	100.0	55	94.8	
Age (years)					
27-39	4	17.4	23	39.7	0.11
40-46	5	21.7	15	25.9	
47-55	8	34.8	9	15.5	
> 55	6	26.1	11	19.0	
Years as a driver					
1-7	5	21.7	18	31.0	0.06
8-15	11	47.8	12	20.7	
16-21	2	8.7	16	27.6	
> 22	5	21.7	12	20.7	
Body mass index					
Healthy weight	1	4.4	7	12.1	0.56
Overweight	11	47.8	27	46.5	
Obesity	11	47.8	24	41.4	
Hours driving					
≤ 8	7	30.4	12	20.7	0.28
8-10	9	39.2	27	46.6	
10-12	7	30.4	11	19.0	
12-16	0	0.0	7	12.1	
> 16	0	0.0	1	1.6	

The following are the adapted variables: rest location, sequence of tasks, lighting, noise, indoor comfort, and thermal comfort.

### REST LOCATION

**Figure 1:**
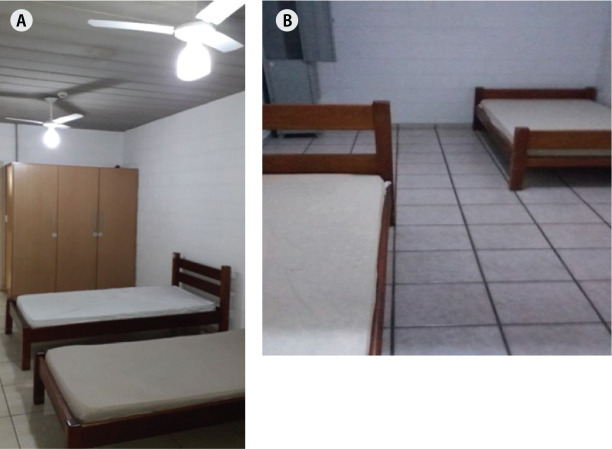
Inside of the bedroom available at the accommodations of branch offices in (A) São Paulo and (B) Campinas.

**Figure 2:**
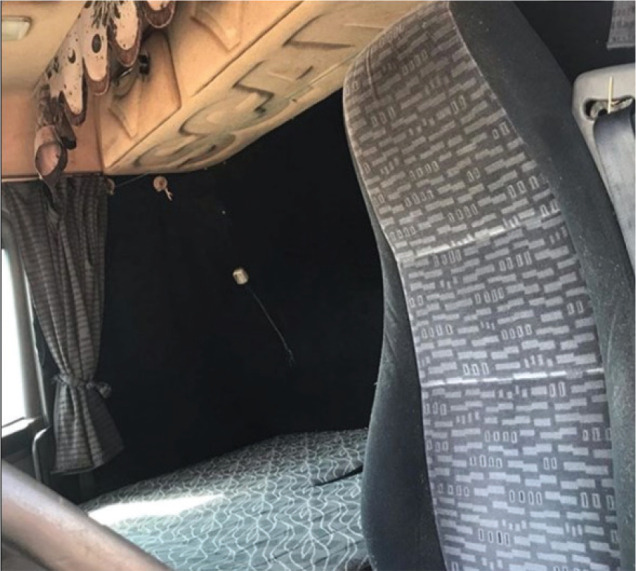
Berth inside one of the drivers’ trucks.

The original EWA suggests a description of the workplace by a schematic diagram of spaces, equipment, materials, and tools. Photography may also be used to improve representation. In this study, this stage was contemplated by photographs of rest locations, as these were the object of study (Figures 1 and 2).

### SEQUENCE OF TASKS

During visits, we were able to observe the workplace dynamics of truck drivers. Long-distance drivers, when arriving from a job, park the vehicle at the corresponding parking space and deliver all shipment documents to the Shipment Processing Center (SPC) of the destination office (São Paulo or Campinas). After checking in, they head to the usual rest locations: accommodations provided by the destination office or the truck sleeper (their own or the company’s).

Drivers working irregular hours (including nighttime) at the long-distance sector usually arrive in the morning, between 08:00 and 12:00, for unloading goods from the origin office. The workday begins between 23:00 and 02:00, and for this reason, assessments were performed mostly at night in both offices. When evaluating the truck, the door and left front window were usually left open, with the engine running. As to the accommodations, assessment were also performed at night, preferably in empty bedrooms or in those where only one bed was taken. Drivers of the daytime shift worked in the morning and afternoon periods.

The truck driver activity begins with a company’s demand for shipment deliveries. The driver should ideally arrive 1 hour before the agreed time in order to verify all procedures required for the preparation and authorization of the trip by the Data Processing Center (DPC). After this authorization, one can proceed with the trip and have the vehicle authorized to leave the company.

The company only allows stops at authorized gas stations, which depending on the itinerary can lead to drivers travelling long distances before taking a break. When the driver arrives at the destination office, he or she should park at the designated parking space and conclude the trip. At this moment, before heading to the rest location, drivers proceed to the dining hall for dinner. After the meal, workers at the São Paulo office can head to the living room, with a television and a sofa, or to their respective rest location (bedroom or truck sleeper). The Campinas office, on the other hand, does not have a living room with television. The drivers thus have the option of heading to their preferred rest location after the meal (bedroom or truck sleeper).

### LIGHTING

**Figure 3:**
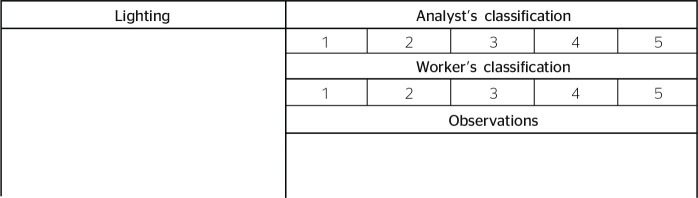
Lighting variable considering the scale of 1 to 5 for subjective evaluation by the analyst and worker.

The lighting assessment, presented in Figure 3, should consider the type of activity performed by workers; lighting was usually measured with a lux meter and the glare level was evaluated by observation. In this study, the assessment was performed subjectively by using the following scale: 1 (very good), 2 (good), 3 (average), 4 (poor), and 5 (very poor). The accommodation and the truck were dark enough for sleeping, because no indirect artificial light (such as light poles) reached the bedroom.

### NOISE

**Figure 4:**
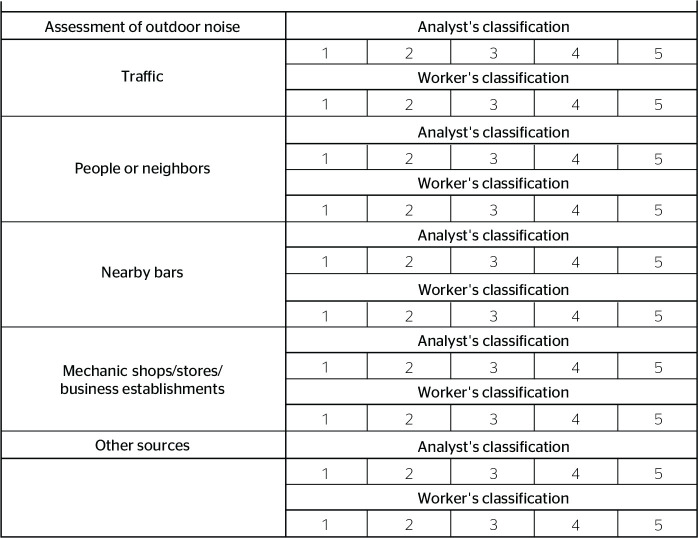
Outdoor noise variable considering a scale ranging from 1 to 5 for subjective evaluation by the analyst and worker.

The assessment of noise at the rest location, as seen in Figure 4, was subjective and divided into indoor and outdoor, being classified in a scale as follows: 1 (no noise), 2 (little noise), 3 (moderate noise), 4 (much noise) e 5 (intolerable noise). For assessing indoor noise, we considered subitems that could affect sleep quality, such as the presence of a roommate or a fan. For assessing outdoor noise, we considered vehicle traffic at the patio, people or neighbors, nearby bars, as well as nearby mechanic shops/stores/business establishments. To both assessments (indoor and outdoor), we added the subitem “others.” It is important to note that, depending on the rest location, some subitems could not be evaluated. For example, when assessing accommodations, the subitem “air conditioning system” was not assessed because it was not available at the São Paulo or Campinas offices.

### INDOOR COMFORT

**Figure 5:**
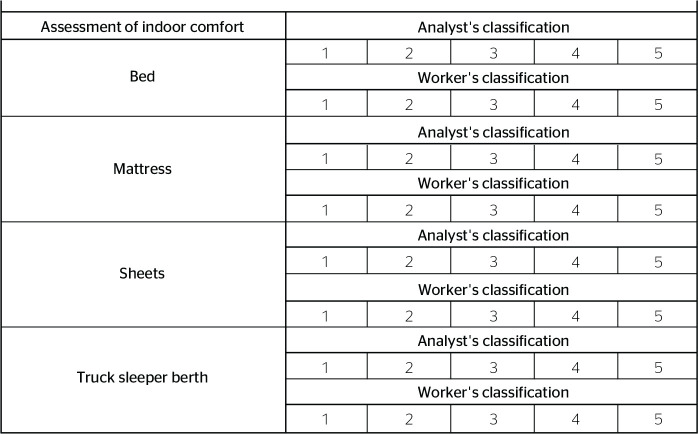
Indoor comfort variable at the rest location considering the scale of 1 to 5 for subjective evaluation by the analyst and worker.

Indoor comfort at the rest location was also subjectively evaluated according to a scale from 1 to 5. The subitems considered in this analysis were bed, mattress, sheets, and truck sleeper berth (Figure 5).

### THERMAL COMFORT

**Figure 6:**
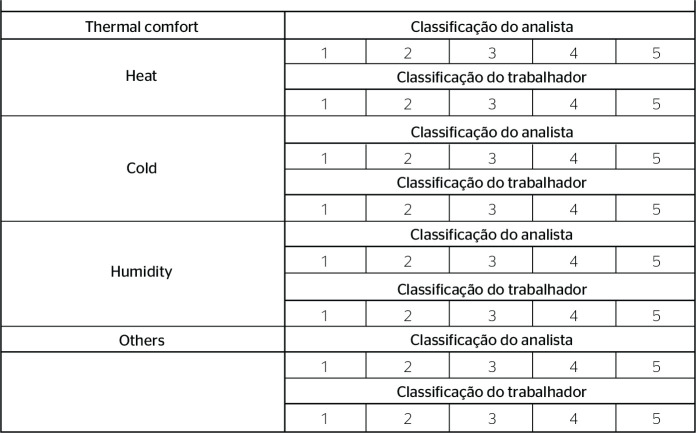
Thermal comfort variable at the rest location considering the scale of 1 to 5 for subjective evaluation by the analyst and worker.

Thermal comfort at the rest location was subjectively evaluated according to a scale from 1 to 5 (Figure 6). The subitems considered in this analysis were heat, cold, and humidity. These subitems were assessed at both locations. The original EWA suggests the assessment of the thermal environment, since the heat load and risks of thermal conditions can compromise occupational health. Other variables mentioned by EWA were not adapted because they could not be measured: general physical activity, lifting, work postures and movements, and risk of accidents.

## DISCUSSION

The use of an instrument based on EWA was shown to be effective owing to the possibility of creating a list of variables that could lead to discomfort in sleep quality at the analyzed rest locations, to be judged both by truck drivers and the analyst.

When considering the worker’s expression in the analysis, the instrument (as EWA) takes advantage of the fact that the worker directly acts or benefits from the work environment (in this case, the rest location), and thus knows its reality. In this context, the fact that this model considers the subjectivity of workers constitutes as an important differential factor in the evaluation of rest locations. However, some items in the original guideline were not used for evaluating rest locations, since the instrument has greater applicability in workplaces involving manual labor or requiring the movement of materials. This way, we sought the expertise of an analyst to adapt the analyzed items. One of the original items that were not used in our analysis is “lifting,” defined as the “worker’s effort to move heavy objects with his or her hands on a non-slippery surface.”

The scale ranging from 1 to 5 was shown to be adequate for evaluating variables, since most drivers understood its meaning from the analyst’s preliminary explanation. This stage took place at the moment of the interview and in detail, that is, each score was orally explained: 1 (very good), 2 (good), 3 (average), 4 (poor), and 5 (very poor). We did not need to alter the number of levels in the scale.

For the subjective assessment of lighting, we considered how much the ambient light in the truck or accommodation could hinder sleep after the trips. The analyses of noise, indoor comfort, and thermal comfort also followed the same principle, always considering the indoor and outdoor spaces at the rest location.

This way, the evaluation instrument was satisfactory and enabled a quantitative, descriptive, and inferential statistical analysis of all items, especially when comparing both assessments. When possible, quantitative descriptive variables were transformed into ordinal variables for tests of proportion.

As to the analysis of the rest location, we observed that this assessment, although practical, was improved through open observations, which are useful for describing the environment. However, according to EWA, other determinant factors of the activity could be investigated, such as activity constraints and strategies and operational modes adopted by drivers; these could then be reunited, systematized, and understood considering the formulation of a pre-diagnosis to guide the systematic observation. At the end of the open analysis, an observation plan is constructed considering the commute of the involved professionals, that is, the meaning of trips to perform a certain activity, the constraints and obstacles to rest.

Some limitations of this study must be noted. One of them was the absence of an analysis of employment bonds and their possible repercussions regarding sleep quality and rest locations. The objective assessment of lighting, noise, and thermal comfort variables can be considered a limitation, although we chose a subjective assessment to avoid distinctions between evaluations by the specialist and the driver, thus obtaining the perception of these variables. Factors such as operational strategies of the activity and its constraints were not investigated, as well as factors of the drivers’ subjectivity and work organization. These pieces of information could complement the evaluation of rest locations. Another limitation of this study concerns the choice of the transportation company for data collection. This is a possible selection bias, since the good working conditions found during visits and the concern with occupational health are among the pillars of the company.

## CONCLUSIONS

This study, by using EWA as the model for an instrument that evaluated truck drivers’ rest locations, showed a new path to be explored in the evaluation of sleep quality and rest locations. The instrument was shown to be adequate for reaching the objectives proposed by this study. We hope that the results obtained in this study are used in new ergonomic approaches and for improving future national policies based particularly on Law No. 13,103, which already clearly states the need for employers to provide adequate rest locations to drivers.
